# Me, myself, and I: self-referent word use as an indicator of self-focused attention in relation to depression and anxiety

**DOI:** 10.3389/fpsyg.2015.01564

**Published:** 2015-10-09

**Authors:** Timo Brockmeyer, Johannes Zimmermann, Dominika Kulessa, Martin Hautzinger, Hinrich Bents, Hans-Christoph Friederich, Wolfgang Herzog, Matthias Backenstrass

**Affiliations:** ^1^Department of General Internal Medicine and Psychosomatics, University Hospital HeidelbergHeidelberg, Germany; ^2^Department of Psychology, University of KasselKassel, Germany; ^3^Institute of Clinical Psychology, Hospital StuttgartStuttgart, Germany; ^4^Department of Clinical Psychology, University of TübingenTübingen, Germany; ^5^Centre for Psychological Psychotherapy, University of HeidelbergHeidelberg, Germany; ^6^LVR-Clinics, Department of Psychosomatic Medicine and Psychotherapy, University Duisburg-EssenEssen, Germany

**Keywords:** self-focused attention, language, pronoun use, depression, chronic depression, anxiety

## Abstract

Self-focused attention (SFA) is considered a cognitive bias that is closely related to depression. However, it is not yet well understood whether it represents a disorder-specific or a trans-diagnostic phenomenon and which role the valence of a given context is playing in this regard. Computerized quantitative text-analysis offers an integrative psycho-linguistic approach that may help to provide new insights into these complex relationships. The relative frequency of first-person singular pronouns in natural language is regarded as an objective, linguistic marker of SFA. Here we present two studies that examined the associations between SFA and symptoms of depression and anxiety in two different contexts (positive vs. negative valence), as well as the convergence between pronoun-use and self-reported aspects of SFA. In the first study, we found that the use of first-person singular pronouns during negative but not during positive memory recall was positively related to symptoms of depression and anxiety in patients with anorexia nervosa with varying levels of co-morbid depression and anxiety. In the second study, we found the same pattern of results in non-depressed individuals. In addition, use of first-person singular pronouns during negative memory recall was positively related to brooding (i.e., the assumed maladaptive sub-component of rumination) but not to reflection. These findings could not be replicated in two samples of depressed patients. However, non-chronically depressed patients used more first-person singular pronouns than healthy controls, irrespective of context. Taken together, the findings lend partial support to theoretical models that emphasize the effects of context on self-focus and consider SFA as a relevant trans-diagnostic phenomenon. In addition, the present findings point to the construct validity of pronoun-use as a linguistic marker of maladaptive self-focus.

## Introduction

The ability to think consciously about oneself in a complex fashion is a unique and generally adaptive psychological aptitude that distinguishes human-beings from other species (Baumeister, 1998; Leary and Buttermore, 2003). However, an excessive and rigid self-focus has been assumed to contribute to health-related problems, particularly to several forms of psychopathology. More specifically, self-focused attention (SFA) is considered a cognitive bias that is closely interconnected with the experience of acute and chronic negative affect (Mor and Winquist, 2002). It is defined as “an awareness of self-referent, internally generated information that stands in contrast to an awareness of externally generated information derived through sensory receptors” (Ingram, 1990, p. 156).

During the last 40 years numerous studies have examined the relation between state and trait SFA and concurrent as well as chronic negative affect. Originally, the construct of SFA was introduced by Duval and Wicklund (1972). According to these authors, SFA initiates a self-evaluative process in which current states in a self-relevant domain are compared to an individual’s ideal states in this domain. Agreement between the actual and ideal states is assumed to result in positive affect whereas negative affect results if the actual state falls short of the ideal state. Carver and Scheier (1998) built on this model and suggested that SFA promotes a self-regulatory cycle which people enter when they experience discrepancies between current and desired states. During this cycle, they are expected to adopt potentially discrepancy-reducing behaviors until the actual state matches the desired state and to continuously rate their progress in achieving the desired state. According to Carver and Scheier, ongoing negative affect is the result of an individual’s judgment that reaching the desired state is unlikely or impossible or that the progress toward the goal is too slow. Pyszczynski and Greenberg (1987) further suggested that individuals who are stuck in this self-regulatory cycle develop a particularly maladaptive style of self-focus in drawing overt attention to themselves after negative events but little attention toward themselves after positive events. This process is assumed to spiral down in an increase of negative internal attributions and self-criticism.

Rumination refers to repetitive and persistent negative thinking that mainly centers around the self (Beck et al., 1979; Teasdale, 1983) and has thus be considered a particular kind of SFA (Mor and Winquist, 2002). According to Nolen-Hoeksema (1991), it is defined as a pattern of responses to distress in which individuals passively and persistently focus on their selves, their symptoms, as well as possible causes and consequences of these symptoms. Rumination is assumed to contribute to the maintenance of negative affect, and depression in particular, through an interference with adaptive coping, goal satisfaction, and emotional processing (Lyubomirsky and Tkach, 2004; Watkins, 2004; Brockmeyer et al., 2014). There is ample evidence that rumination predicts the onset of depression (Just and Alloy, 1997; Nolen-Hoeksema, 2000) and future levels of depression, both in clinical and non-clinical samples (Aldao et al., 2010). More recently, the construct of rumination has been subdivided into two distinct components, termed brooding and reflection (Treynor et al., 2003). Whereas reflection refers to a “purposeful turning inward to engage in cognitive problem solving to alleviate one’s depressive symptoms,” brooding refers to the passive comparison of one’s current state with desired but unreached states (Treynor et al., 2003, p. 256). Previous research suggests that brooding represents the more maladaptive of these two subcomponents (Joormann et al., 2006; Raes and Hermans, 2008; Cox et al., 2012; Gooding et al., 2012).

Self-focused attention has been mostly discussed in the context of depression. Meta-analyses found medium to large cross-sectional associations between various measures of SFA and depression (Mor and Winquist, 2002; Aldao et al., 2010). However, a lively debate has risen in the 1990s centering around the question whether SFA relates to negative affect in general or only to specific disorders. Some authors have argued that it is specific to depression (Pyszczynski et al., 1991) whereas others have argued that it rather constitutes a general factor across different forms of psychopathology (Ingram, 1990). For example, with anxiety it has been assumed that threatening experiences may increase a person’s self-focus and the person’s impression that it is impossible to cope with these experiences which in turn may trigger avoidance behavior which then further increases anxiety (Carver and Blaney, 1977; Carver et al., 1979). In support of the trans-diagnostic view of SFA, Mor and Winquist (2002) found in their meta-analysis that SFA was positively related with both symptoms of depression and anxiety across studies, although the link between SFA and depression was stronger. Across the different sub-dimensions of anxiety, generalized anxiety symptoms showed the strongest relationship with SFA. Furthermore, SFA may even be associated with mental disorders other than depression and anxiety. For instance, many symptoms of eating disorders (e.g., the preoccupation with one’s body shape and weight) could be interpreted as excessive SFA (Zucker et al., 2014).

In his influential yet controversial model of chronic depression, McCullough (2000, 2003) has argued that chronically depressed patients, particularly those with an early onset of the disorder, feature a particularly pronounced self-focus (i.e., egocentricity) due to a distinct deficit in socio-emotional processing stemming from early adverse experiences with significant others and an insufficient maturation. According to McCullough, this self-centeredness involves an insufficient attention to the emotional states of others and a disconnection from social feedback. However, studies examining this postulated deficit in chronic depression are scarce and have so far yielded inconsistent results. Whereas one study found such deficits in chronically depressed patients as compared to healthy controls (Zobel et al., 2010), two other studies found no such impairments (Wilbertz et al., 2010; van Randenborgh et al., 2012). Worthy of note, only one out of these three studies compared chronically and non-chronically depressed patients in this manner and found no group differences (van Randenborgh et al., 2012). However, these studies uniformly focused on one (major) aspect of the proposed egocentricity in chronic depression: the lack of empathy and understanding of emotional states in others. To the best of our knowledge, however, no study has yet examined the self-centered attention itself in chronic and non-chronic depression. Thus, one of the aims of the present study was to compare chronically, non-chronically, and never-depressed individuals in this realm.

Previous studies examining individual differences in SFA have mostly relied on self-report measures and sentence completion tasks (Mor and Winquist, 2002). However, linguistic analyses offer a non-obtrusive, objective measure of spontaneously occurring SFA (Pennebaker et al., 2003; Tausczik and Pennebaker, 2010). Tracking an individual’s language use provides information on what the individual is attending to. Particularly, the relative frequency with which a person refers to him- or herself in written or spoken language reflects his or her degree of SFA (Tausczik and Pennebaker, 2010). Research in experimental social psychology suggests that the use of first-person singular pronouns is closely related to current SFA. For instance, first-person singular pronouns were used as a manipulation check in experiments in order to prove whether the manipulation of self-focus (e.g., a mirror in front of the participant) was successful (Davis and Brock, 1975; Salovey, 1992). Moreover, writing essays containing first-person singular pronouns has itself been successfully used as a manipulation of SFA (Fenigstein and Levine, 1984; Pyszczynski et al., 1987). Similar to research using self-reports of SFA, previous studies have repeatedly demonstrated a positive relationship between the use of first-person singular pronouns in written and spoken language and depression (Rude et al., 2004; Sloan, 2005; Mehl, 2006; Fast and Funder, 2010; Molendijk et al., 2010; Zimmermann et al., 2013).

Several authors have suggested that SFA is not maladaptive *per se* but only under certain conditions and in certain contexts (Duval and Wicklund, 1972; Pyszczynski and Greenberg, 1987; Carver and Scheier, 1998). As described above, most theoretical models assume that SFA becomes dysfunctional if an individual experiences a negative discrepancy between a current and a desired personal state. Such discrepancies are more likely to occur in the face of negative events such as losses or failures. It appears plausible that increased SFA following negative but not following positive events leads to increased negative affect (Pyszczynski and Greenberg, 1987). Accordingly, several studies have shown that experimentally induced sad mood led to increased SFA whereas neutral and happy moods did not (Wood et al., 1990; Sedikides, 1992). Correspondingly, in their meta-analysis on SFA and negative affect, Mor and Winquist (2002) found that elevated SFA after a negative but not after a positive event was associated with negative affect.

Taken together, the theoretical models and empirical evidence outlined above suggests that SFA in the context of negative but not positive events is associated with symptoms of depression and anxiety, and more strongly with depression. However, previous studies examining pronoun use as an indicator of SFA have mainly focused on non-clinical samples (Rude et al., 2004; Sloan, 2005; Mehl, 2006; Fast and Funder, 2010). Studies on language use and depression in clinical populations examined either patients with personality disorders and comorbid depression (Molendijk et al., 2010), mixed clinical and non-clinical samples involving patients with different disorders (Zimmermann et al., 2013), or very small samples (Bucci and Freedman, 1981). Additionally, there is a lack of studies examining the relation between SFA and depression and anxiety at the same time. Moreover, studies examining SFA in a positive versus a negative context have relied solely on self-reports and we are not aware of such a study in a clinical sample. In sum, no study has yet examined whether linguistic markers of spontaneously occurring SFA in the context of negative versus positive events are associated with symptoms of (chronic and non-chronic) depression and anxiety in clinical and non-clinical samples. In addition, there is a lack of studies investigating the convergence of self-reported and objectively measured SFA. The two studies presented here aimed to overcome these gaps in the literature and thus to contribute to the understanding of the nexus between SFA and psychopathology.

*Hypotheses:* Based on the theoretical models and empirical evidence outlined above we expected the following:

(1) First-person singular pronoun use during the recall of a negative (1a) but not during the recall of a positive (1b) autobiographical event will be positively related to symptoms of depression and anxiety in both a non-clinical and three different clinical samples.
(2) Across the different samples, first-person singular pronoun use during negative memory recall will be more strongly associated with symptoms of depression than with symptoms of anxiety.
(3) Both patients with chronic and those with episodic depression will use more first-person singular pronouns than healthy, never-depressed controls during negative but not during positive memory recall (3a). Chronically depressed patients will use even more first-person singular pronouns than non-chronically depressed patients during negative but not during positive memory recall (3b).
(4) Across samples, first-person singular pronoun use during negative memory recall will be positively related to brooding (representing the more maladaptive component of ruminative self-focus) but not with reflection.

## Study 1

As described above, SFA does not limit to depression only but also shows relations to other mental disorders. For instance, many of the symptoms of anorexia nervosa can be interpreted in a fashion of disturbed SFA. The characteristic preoccupation with body shape and weight points to a strongly narrowed attention toward the self in this population. Indeed, a recent study has shown elevated levels of self-reported SFA in people with a history of anorexia nervosa (Zucker et al., 2014). Furthermore, depression and anxiety, the two symptom clusters that have been most commonly discussed in the context of SFA, are both frequently comorbid with anorexia nervosa at full-syndrome and sub-threshold levels (Wade et al., 2000; Mattar et al., 2011). There is some evidence pointing into the direction that malnutrition and resulting underweight in anorexia nervosa cause several symptoms of depression and anxiety such as sleep disturbances, agitation, loss of energy, and decreased abilities to think or concentrate (Mattar et al., 2012; Gauthier et al., 2014). Moreover, comorbid depression was found to predict a less favorable course of the disorder (Löwe et al., 2001; Fichter et al., 2006). Based on these findings, it appears reasonable and clinically relevant to examine SFA and its association with symptoms of depression and anxiety in this clinical population.

### Materials and Methods

#### Participants

Twenty-five patients with anorexia nervosa, recruited from the inpatient units of a university hospital, took part in the first study. The sample has been described in detail elsewhere (Brockmeyer et al., 2013). However, data on self-referent word use from this sample have not been analyzed previously. All participants were female, Caucasian, and between 18 and 45 years of age. Participants were only included if they met the criteria for anorexia nervosa according to the Diagnostic and Statistical Manual of Mental Disorders, Fourth Edition (American Psychiatric Association [APA], 1994) at the time of the study and if they did not meet one of the following exclusion criteria: current or life-time diagnosis of a manic episode, psychosis, or borderline personality disorder. Written informed consent was obtained from all participants, and the study was approved by the local ethics committee.

#### Measures

*Symptoms of depression and anxiety* were assessed with the Patient Health Questionnaire-9 (PHQ-9; Gräfe et al., 2004) and the Generalized Anxiety Disorder Screener-7 (GAD-7; Löwe et al., 2008). The items of both the PHQ-9 and the GAD-7 reflect the DSM-IV criteria of major depression and generalized anxiety disorder, respectively (American Psychiatric Association [APA], 1994). On both instruments participants are asked to rate how often, during the last 2 weeks, they have suffered from each of these symptoms. Response options are *not at all*, *several days*, *more than half the days*, and *nearly every day*. Total scores range from 9 to 27 in the PHQ-9, and from 0 to 21 in the GAD-7. Both instruments are commonly used, economic self-reports that have repeatedly shown excellent psychometric properties in previous studies (Löwe et al., 2004a,b; Spitzer et al., 2006).

*First-person singular pronoun use* was considered as an indicator of SFA and was measured by computerized text analysis of written essays from an autobiographical memory recall task. Referring to the *Emotions Interview* developed by Rottenberg et al. (2006), participants were instructed to describe one of the saddest and one of the happiest moments in their lives and were provided with themes that often exemplify those events. Participants were guided by several standardized probe questions on their answer sheets to facilitate elaboration on the emotional quality of the events (e.g., “Can you describe why this event made you feel sad/happy?”, “As you think about this sad/happy event now, what thoughts or feelings come to mind?”). The reports of each participant were then analyzed with a computerized text analysis software, the Linguistic Inquiry and Word Count (LIWC; Pennebaker et al., 2001). LIWC uses a word count strategy and calculates the relative frequency of words and word stems in a text that fall into a specific category such as first-person singular pronouns (e.g., “I”, “me”, “mine”). Previous studies have demonstrated the temporal stability of language use across time, topic, and text source, even within short time frames (Gleser et al., 1959; Schnurr et al., 1986; Pennebaker and King, 1999).

### Results

#### Sample Characteristics

The sample was composed of 20 patients with anorexia nervosa of the restricting subtype and five of the binge/purge subtype. Out of the 25 patients, six had a comorbid MDD, one had a comorbid obsessive-compulsive disorder, and one had a comorbid MDD and a comorbid anxiety disorder as well. Descriptive data are summarized in **Table 1**.

**Table 1:** Descriptive data for the samples of Study 1 and 2.

	Study 1	Study 2		
	
Characteristic	Patients with anorexia nervosa (*n* = 25)	Patients with chronic depression (*n* = 29)	Patients with non-chronic depression (*n* = 30)	Healthy controls (*n* = 29)
Gender				
% Women	100	62	67	60
% Inpatients	100	66	80	–
Mean age in years (*SD*)	24.64 (7.50)	39.17 (11.68)	38.57 (11.44)	38.33 (14.54)
Co-morbid diagnoses Major depressive disorder (cases) Anxiety disorder (cases) Eating disorder (cases) Obsessive-compulsive disorder (cases)	6 0 – 1	– 8 2 2	– 8 1 0	– – – –
% Currently on psychotropic medication	0	69	77	0
% Currently in psychotherapy	100	79	77	0
Mean BDI-II score (*SD*)^∗^	–	26.00 (10.87)^a^	23.45 (9.64)^a^	2.17 (2.93)^b^
Mean PHQ-9 score (*SD*)	11.36 (5.45)	–	–	–
Mean GAD-7 score (*SD*)^∗^	8.00 (3.52)	8.69 (4.73)^a^	8.90 (5.16)^a^	1.50 (2.60)^b^
Mean RSQ-Brooding score (*SD*)^∗^	–	12.59 (3.86)^a^	12.90 (3.41)^a^	7.50 (1.41)^b^
Mean RSQ-Reflection score (*SD*)^∗^	–	11.48 (3.97)^a^	12.07 (3.27)^a^	6.17 (1.42)^b^
Mean Pronoun use in positive memory task (*SD*)^∗∗^	10.22 (3.27)	7.16 (2.81)	7.50 (2.68)	6.43 (3.21)
Mean pronoun use in negative memory task (*SD*)	10.66 (4.04)	8.02 (2.10)^a^	8.91 (2.95)^a^	6.85 (3.44)^b^

*DI-II, Beck Depression Inventory-II; PHQ-9, Patient Health Questionnaire-9 (Depression Module); GAD-7, General Anxiety Disorder Screener-7; RSQ, Responses Style Questionnaire (Ruminative Response Scale); Pronoun use, relative frequency of first-person singular pronouns in percent; ^∗^data were missing from one participant; ^∗∗^positive memory data were only available from 19 patients with anorexia nervosa; different superscripts denote group differences.*

#### Associations between Pronoun Use and Symptoms of Depression and Anxiety

In order to examine the hypothesized associations between pronoun use and psychopathology (Hypotheses 1A,B), Pearson correlation coefficients were calculated. As displayed in **Table 2**, the relative frequency of first-person singular pronouns during negative memory recall was positively correlated with symptoms of depression and anxiety whereas pronoun use during positive memory recall was not.

**Table 2:** Correlations between pronouns use, depression, anxiety, and rumination in the different subsamples.

	Symptoms of depression	Symptoms of anxiety	Brooding-rumination	Reflection-rumination
Pronoun use during negative memory recall	*r* = 0.461, *p* = 0.020 (AN)	*r* = 0.545, *p* = 0.005 (AN)	Not assessed	Not assessed


	*r* = 0.577, *p* = 0.013 (ND)	*r* = 0.787, *p* < 0.001 (ND)^∗^	*r* = 0.469, *p* = 0.010 (ND)	*r* = 0.108, *p* = 0.578 (ND)
	*r* = -0.032, *p* = 0.809 (MDD)^∗^	*r* = 0.001, *p* = 0.993 (MDD)	*r* = 0.177, *p* = 0.184 (MDD)^∗^	*r* = 0.093, *p* = 0.486 (MDD)^∗^
Pronoun use during positive memory recall	*r* = 0.025, *p* = 0.919 (AN)^∗∗^	*r* = 0.087, *p* = 0.725 (AN)^∗∗^	Not assessed	Not assessed


	*r* = 0.235, *p* = 0.220 (ND)	*r* = 0.326, *p* = 0.091 (ND)^∗^	*r* = 0.053, *p* = 0.787 (ND)	*r* = 0.128, *p* = 0.508 (ND)
	*r* = 0.183, *p* = 0.168 (MDD)^∗^	*r* = 0.004, *p* = 0.979 (MDD)	*r* = 0.093, *p* = 0.488 (MDD)^∗^	*r* = -0.046, *p* = 0.734 (MDD)^∗^

*Pronoun use = relative frequency of first-person singular pronouns in percent; Symptoms of depression were assessed with the Depression Module of the Patient Health Questionnaire (PHQ-9) in patients with anorexia nervosa and with the Beck Depression Inventory-II (BDI-II) in the other subsamples; Symptoms of anxiety were assessed using the General Anxiety Disorder Screener (GAD-7); Brooding and Reflection were assessed using the Ruminative Response Scale of the Responses Style Questionnaire (RSQ); AN = Patients with anorexia nervosa (n = 25); ND = Healthy, never-depressed participants (n = 29); MDD = Patients with major depressive disorder (n = 59); ^∗^data were missing from 1 participant; ^∗∗^positive memory recall data were only available from 19 patients with anorexia nervosa.*

To examine whether the association between pronoun use and depression was stronger than the association between pronoun use and anxiety (Hypothesis 2), each correlation was converted into a *z*-score using Fisher’s *r*-to-*z* transformation (Fisher, 1921). Subsequently, the asymptotic covariance of the estimates was calculated as described by Steiger (1980) and used in an asymptotic *z*-test. This revealed that the strengths of the two correlations did not differ significantly, *z* = 0.62, *p* = 0.534.

### Summary of Study 1

The results of Study 1 support Hypotheses 1a and ab inasmuch pronoun use during negative but not positive memory recall was positively correlated with symptoms of depression and anxiety. Hypothesis 2 was not supported since pronoun use was not significantly stronger correlated with depression than with anxiety symptoms. Hypotheses 3 and 4 were not tested in Study 1.

## Study 2

The second study aimed to further examine the associations between SFA in a positive and a negative context and symptoms of depression and anxiety in two samples, (a) healthy, never-depressed individuals from the general population and (b) patients with a major depressive disorder (MDD). Furthermore, we aimed to investigate the convergence between pronoun use and self-reported aspects of SFA by also assessing two facets of rumination in these samples.

### Materials and Methods

#### Participants

Participants for Study 2 were obtained from a larger project that has been described elsewhere (Brockmeyer et al., 2015). Data on self-referent word use and rumination from this sample, however, have not been analyzed previously. From two participants of the original sample no autobiographic memory data was available. Thus, the total sample for the present analyses was composed of 29 patients with chronic depression, 30 patients with non-chronic depression, and 29 healthy, never-depressed controls. Patients were recruited from the department of psychiatry of a community hospital and from a large outpatient psychotherapy center. Healthy controls were recruited via advertisements in the local media and from a university campus. Participants were interviewed by trained clinicians using the *Structured Clinical Interview for DSM-IV Axis I and II* (SCID; Wittchen et al., 1997).

To be included, all participants had to be between 18 and 60 years old. In addition, participants in the episodic depression group had to meet the DSM-IV criteria of a current MDD, and participants in the chronic depression group had to feature either (a) a chronic (≥2 years) course of a single episode of MDD, or (b) an insufficient remission between several episodes of a MDD that have lasted at least 2 years in total, or (c) a so-called double depression (i.e., single or recurrent episodes of MDD and comorbid dysthymic disorder) for at least 2 years. Because previous research has found that early onset (at <21 years of age) chronic depression is associated with a more malignant course than the late onset form (Klein et al., 1999a,b) and because particularly the early onset form has been assumed to be related with an elevated self-focus (McCullough, 2000), we further specified that only participants with an early onset were eligible for the chronic depressed group.

Exclusion criteria for the two patient groups were: comorbid substance abuse or dependence, a lifetime diagnosis of bipolar disorder, or psychosis, according to the DSM-IV. Exclusion criteria for the healthy control group were: a current mental disorder according to the DSM-IV or a lifetime diagnosis of depression, bipolar disorder, or psychosis. All participants received financial compensation. Patients were assessed prior to or at the beginning of their treatment. Written informed consent was obtained from all participants, and the study was approved by the local ethics committee.

#### Measures

*Symptoms of depression* were measured using the *Beck Depression Inventory-II* (BDI-II; Beck et al., 1996; Kühner et al., 2007). *Symptoms of anxiety* were assessed with the GAD-7 (Löwe et al., 2008) like in Study 1. *Rumination* was measured using the 10-item short form of the *Ruminative Responses Scale of the Response Styles Questionnaire* (RSQ; Huffziger and Kuhner, 2012). This version of the RSQ allows for the differentiation of the two subcomponents brooding and reflection, each measured by five items.

*First-person singular pronoun use* was assessed with a similar method as in Study 1. The only difference was that participants did not write essays but where personally interviewed on a positive and a negative autobiographic memory. However, the interview followed the same structure like the writing instructions in Study 1 and the same probe questions were used to activate the memories. The complete interview was recorded on audio and transcribed into Standard German. Only participant speech was analyzed, interviewer speech was deleted instead. Again, the LIWC was used to determine the proportion of first-person singular pronouns in the participants’ speech.

### Results

#### Sample Characteristics

Means and standard deviations of all variables are summarized in **Table 1**. The groups did not differ in terms of age, *F*(2,85) = 0.02, *p* = 0.976, and gender, χ^2^(2) = 0.41, *p* = 0.814. However, as to be expected, groups differed regarding symptoms of depression, *F*(2,84) = 67.80, *p* < 0.001, and anxiety *F*(2,84) = 26.86, *p* < 0.001, as well as in terms of brooding, *F*(2,84) = 28.62, *p* < 0.001, and reflection, *F*(2,84) = 33.10, *p* < 0.001. *Post hoc* comparisons showed that the two depressed groups scored higher than healthy controls in all four scales (all *p*s < 0.001) but did not differ from each other (all *p*s > 0.259).

#### Group Differences Regarding Pronoun Use

In order to examine context-specific group differences in pronoun use (Hypotheses 3a and 3b), a 3 × 2 repeated measures ANOVA was conducted with Group (chronically depressed, non-chronically depressed, healthy controls) as between-subject factor and Context (positive versus negative memory recall) as within-subject factor. The analysis revealed a significant main effect for Group, *F*(2,85) = 3.21, *p* = 0.045, and a significant main effect for Context, *F*(1,85) = 6.54, *p* = 0.012, but no significant Group × Context interaction effect, *F*(2,85) = 0.69, *p* = 0.506. *Post hoc* pairwise comparisons showed only one significant group difference, i.e., that non-chronically depressed patients used generally more first-person singular pronouns than healthy controls, *p* = 0.014 (Cohen’s *d* = 0.36 and 0.64 in the positive and the negative context, respectively).

#### Associations between Pronoun Use, Symptoms of Depression and Anxiety, and Rumination in Healthy Participants

In order to examine the associations between first-person singular pronoun use, psychopathology, and rumination in the healthy control group, again Pearson correlation coefficients were calculated (see **Table 2**). Frequency of first-person singular pronouns during negative memory recall was positively correlated with symptoms of depression and anxiety (Hypotheses 1a and 1b) as well as with brooding but not with reflection (Hypothesis 4). Frequency of first-person singular pronouns during positive memory recall instead, was not correlated with any of these variables.

With respect to Hypothesis 2, the strength of the correlation between pronoun use during negative memory recall and symptoms of depression did not differ significantly from the strength of the correlation between pronoun use during negative memory recall and symptoms of anxiety, *z* = 1.82, *p* = 0.069.

#### Associations between Pronoun Use and Symptoms of Depression and Anxiety in Depressed Patients

Since the two depressed patient groups did not differ in any of the variables of interest (i.e., symptoms of depression and anxiety, brooding, reflection, and pronoun use during positive and negative memory recall) and for the sake of comprehensibility, they were treated as one group in the following last part of analyses. Analogous to the analyses in the healthy control group, Pearson correlation coefficients were calculated to examine Hypotheses 1a and 1b as well Hypothesis 4. As can be seen in **Table 2**, pronoun use in this subsample was not correlated with depression, anxiety, and rumination, neither during negative memory recall nor during positive memory recall.

### Summary of Study 2

The findings of Study 2 provide partial support for Hypotheses 1a and 1b since pronoun use in a negative, but not in a positive context, was positively related to symptoms of depression and anxiety in healthy, never-depressed participants but not in the clinical sample of patients with a MDD. Hypothesis 2 was not supported since the strengths of the correlations between pronoun use and depression and pronoun use and anxiety did not differ significantly. Hypothesis 3a and 3b found only partial support inasmuch non-chronically depressed patients used generally more first-person singular pronouns (irrespective of context) than healthy controls, and all groups (irrespective of diagnostic status) used more first-person singular pronouns during negative than during positive memory recall. Contrary to the theoretical models described above, patients with chronic depression did not use more first-person singular pronouns than patients with non-chronic depression and healthy controls. Hypothesis 4 received also partial support in that pronoun use during negative, but not positive, memory recall was positively correlated with self-reported brooding but not with reflection in healthy, never-depressed participants. However, this effect was again specific to the sample of healthy participants and could not be observed in the two samples of depressed patients.

## General Discussion

The present studies are the first to examine SFA in a positive and negative context in patients with a MDD, patients with anorexia nervosa, and healthy, never-depressed individuals using an objective, language-based measure. We found that first-person singular pronoun use (as an indicator of SFA) during the recall of a negative, but not during the recall of a positive, autobiographical memory was positively related to symptoms of depression and anxiety in patients with anorexia nervosa and in healthy individuals. These findings extend previous studies that showed positive correlations between first-person singular pronoun use and self-reported symptoms of depression in undergraduates (Mehl, 2006; Fast and Funder, 2010). The present findings further support theoretical models according to which SFA is particularly maladaptive after negative but not after positive events (Pyszczynski and Greenberg, 1987). This corresponds to previous findings that experimentally induced sad mood leads to increased SFA whereas neutral and happy moods do not (Wood et al., 1990; Sedikides, 1992).

Further pointing to the construct validity of first-person singular pronoun use as an indicator of maladaptive self-focus, it was positively correlated with brooding but not with reflection in healthy individuals. However, the pattern of correlations between pronoun use during negative memory recall, symptoms of depression and anxiety, and brooding could not be observed in the two samples of depressed patients. In partial support of our hypotheses, however, non-chronically depressed patients used more first-person singular pronouns than healthy, never-depressed controls. This latter finding is in line with previous findings of greater first-person singular pronoun use in dysphoric as compared to non-dysphoric undergraduate students (Rude et al., 2004; Sloan, 2005) as well as in depressed as compared to non-depressed patients (Bucci and Freedman, 1981; Molendijk et al., 2010). The present study extends this previous work by examining pronoun use in larger samples of patients with a primary diagnosis of MDD in both a positive and a negative context. Nevertheless, we were not able to demonstrate a context-specific elevation of self-focus in depressed patients during negative memory recall. Instead, depressed patients used more first-person singular pronouns, independent of context, and both depressed patients and healthy controls used more first-person singular pronouns during negative than during positive memory recall. Contrary to our hypotheses, this pattern of findings suggests a greater general self-focus in clinical depression, irrespective of context, and a general self-focus increasing effect of negative mood, irrespective of clinical status.

Importantly, based on the specific operationalization of self-focus that we have applied in the present study, the present findings do not support McCullough’s theoretical model of pronounced self-focus in early onset chronic depression (McCullough, 2000, 2003). Corresponding to previous empirical studies that did not find a lack of empathy or theory of mind in patients with chronic depression (Wilbertz et al., 2010; van Randenborgh et al., 2012), the present findings challenge this concept of chronic depression and call for a reconceptualization.

When reviewing the literature on pronoun use and depression, it becomes apparent that correlations between first-person singular pronoun use and symptoms of depression were only found in non-clinical or mixed clinical and non-clinical samples (Mehl, 2006; Fast and Funder, 2010; Zimmermann et al., 2013). Although there seem to be robust effects showing that depressed patients use more first-person singular pronouns than non-depressed individuals (Rude et al., 2004; Sloan, 2005; Molendijk et al., 2010), no study could yet observe a significant correlation between pronoun use and levels of depressive symptoms in currently depressed samples (Molendijk et al., 2010). Taken together, these findings suggest that elevated use of first-person singular pronouns may rather contribute to mild and moderate symptoms of depression but is less crucial in acute phases of full-syndrome MDD. Alternatively, ceiling effects in depression measures could have limited the ability to detect linear relationships between the degree of self-focus and symptom severity in clinical samples of depressed patients.

Finally, the present findings do not clearly support the specificity of SFA for depression. In patients with anorexia nervosa and in healthy controls, pronoun use during negative memory recall was significantly correlated with symptoms of depression and anxiety. In both samples, pronoun use did not show a significantly stronger relation to depression than to anxiety symptoms. Although Mor and Winquist (2002) meta-analytically found a stronger association between SFA and depression than between SFA and anxiety across studies, they reported significant effect sizes for the link between SFA and anxiety, particularly generalized anxiety, as well. The present findings of comparable associations between pronoun use and depression and anxiety are in line with those theoretical models that stress SFA as a trans-diagnostic factor that appears also relevant for the development and/or maintenance of anxious mood states (Carver and Blaney, 1977; Carver et al., 1979; Ingram, 1990).

### Treatment Implications

The present findings may also have some treatment implications as they suggest that (a) targeting maladaptive self-focus may be particularly helpful with mild to moderate levels of depression, and (b) that targeting SFA may also be suitable in the treatment of anxiety and eating disorders. However, previous research suggests that SFA in the form of rumination typically does not change significantly in response to standard cognitive-behavioral therapy (CBT; Watkins and Nolen-Hoeksema, 2014). Instead, rumination-focused CBT that incorporates functional analysis and stimulus control has been shown to effectively reduce rumination and depression (Watkins et al., 2011). Similarly, there is preliminary evidence that rumination-focused treatment packages that foster externally oriented attention, question positive metacognitive beliefs about seeming advantages of rumination, and involve expressive writing and training in problem-solving can reduce levels of rumination (Teismann et al., 2014). In addition, previous clinical trials have shown that writing itself can reduce maladaptive SFA as indexed by rumination when adopting certain strategies during the writing process such as focusing on current emotions and feelings and relating the experiences one has made to other aspects of one’s life (Sloan et al., 2008). Finally, Bond and Pennebaker (2012) demonstrated how a specific writing instruction that was tailored to the individual’s actual pronoun use and provided during a brief, standardized writing assignment successfully induced a change of pronoun use.

### Limitations and Future Directions

Several limitations of the present studies should be noted. First, the sample sizes were relatively small, resulting in limited statistical power. We cannot rule out that there are small to moderate effects in the population which we were not able to detect. Thus, the findings need further testing in larger samples. Second, both studies presented here used a cross-sectional design, limiting any causal conclusions. We therefore cannot draw any conclusions whether pronoun use represents a precursor or a result of psychopathology. Prospective, longitudinal studies are needed that examine the role of pronoun use as an indicator of self-focused attention in the etiology of mental disorders. Finally, future studies on language and psychopathology may not only use quantitative text analysis as applied by the LIWC but also other methods like n-grams and topic models that could help to reveal linguistic structures associated with depression and anxiety (Imel et al., 2015).

## Conclusion

Using an unobtrusive, objective measure of spontaneously emerging SFA in written and spoken language during the recall of positive and negative autobiographic events, the present study unraveled some important insights into the link between first-person singular pronoun use and symptoms of depression and anxiety. The present findings add to the literature by demonstrating an effect of context on the link between self-focus and psychopathology as only first-person singular pronoun use during negative memory recall but not during positive memory recall was associated with symptoms of depression and anxiety in patients with anorexia nervosa and in healthy controls. Furthermore, the present findings speak in favor of SFA as a trans-diagnostic phenomenon since pronoun use was equally related to symptoms of depression and anxiety. Although, this pattern of associations could not be observed in the samples of clinically depressed patients, the result of generally increased first-person pronoun use in depressed as compared to never-depressed participants underscores the relevance of self-focus also in clinical depression. Finally, the present findings close a gap in the literature by showing for the first time that first-person pronoun use during negative but not positive memory recall is positively related to brooding which is considered the more maladaptive subcomponent of ruminative self-focus.

## Author Contributions

TB was responsible for the design of the study, for data acquisition, analysis, and interpretation, and for writing the first draft of the manuscript. JZ contributed to the design of the work, the analysis and interpretation of the data, and the revision of the manuscript. DK contributed to data acquisition and helped revising the manuscript. HB, HCF, WH, and MB helped with data acquisition and interpretation and helped to revise the manuscript. MH helped with the interpretation of the data and the revision of the manuscript. All authors approved the final version of the manuscript and agreed to be accountable for all aspects of the work.

## Conflict of Interest Statement

The authors declare that the research was conducted in the absence of any commercial or financial relationships that could be construed as a potential conflict of interest.
